# Droplet digital PCR for the quantification of Alu methylation status in hematological malignancies

**DOI:** 10.1186/s13000-018-0777-x

**Published:** 2018-12-22

**Authors:** Paola Orsini, Luciana Impera, Elisa Parciante, Cosimo Cumbo, Crescenzio F. Minervini, Angela Minervini, Antonella Zagaria, Luisa Anelli, Nicoletta Coccaro, Paola Casieri, Giuseppina Tota, Claudia Brunetti, Alessandra Ricco, Paola Carluccio, Giorgina Specchia, Francesco Albano

**Affiliations:** Department of Emergency and Organ Transplantation (D.E.T.O.), Hematology Section, University of Bari, P.zza G. Cesare, 11 70124 Bari, Italy

**Keywords:** Alu repeats, DNA methylation, ddPCR, Hematological malignancies, Hypomethylating agents

## Abstract

**Background:**

Alu repeats, belonging to the Short Interspersed Repetitive Elements (SINEs) class, contain about 25% of CpG sites in the human genome. Alu sequences lie in gene-rich regions, so their methylation is an important transcriptional regulation mechanism. Aberrant Alu methylation has been associated with tumor aggressiveness, and also previously discussed in hematological malignancies, by applying different approaches. Moreover, today different techniques designed to measure global DNA methylation are focused on the methylation level of specific repeat elements.

In this work we propose a new method of investigating Alu differential methylation, based on droplet digital PCR (ddPCR) technology.

**Methods:**

Forty-six patients with hematological neoplasms were included in the study: 30 patients affected by chronic lymphocytic leukemia, 7 patients with myelodysplastic syndromes at intermediate/high risk, according with the International Prognostic Scoring System, and 9 patients with myelomonocytic leukemia. Ten healthy donors were included as controls. Acute promyelocytic leukemia-derived NB4 cell line, either untreated or treated with decitabine (DEC) hypomethylating agent, was also analyzed.

DNA samples were investigated for Alu methylation level by digestion of genomic DNA with isoschizomers with differential sensitivity to DNA methylation, followed by ddPCR.

**Results:**

Using ddPCR, a significant decrease of the global Alu methylation level in DNA extracted from NB4 cells treated with DEC, as compared to untreated cells, was observed. Moreover, comparing the global Alu methylation levels at diagnosis and after azacytidine (AZA) treatment in MDS patients, a statistically significant decrease of Alu sequences methylation after therapy as compared to diagnosis was evident. We also observed a significant decrease of the Alu methylation level in CLL patients compared to HD, and, finally, for CMML patients, a decrease of Alu sequences methylation was observed in patients harboring the SRSF2 hotspot gene mutation c.284C>D.

**Conclusions:**

In our work, we propose a method to investigate Alu differential methylation based on ddPCR technology. This assay introduces ddPCR as a more sensitive and immediate technique for Alu methylation analysis. To date, this is the first application of ddPCR to study DNA repetitive elements. This approach may be useful to profile patients affected by hematologic malignancies for diagnostic/prognostic purpose.

**Electronic supplementary material:**

The online version of this article (10.1186/s13000-018-0777-x) contains supplementary material, which is available to authorized users.

## Background

DNA methylation is an epigenetic modification occurring at 5′cytosine of CpG dinucleotides; it plays a pivotal role in genome regulation in several physiological processes such as genomic imprinting, X inactivation and hematopoietic differentiation [1]. Variations of DNA methylation contribute to tumorigenesis and tumor maintenance, and aberrant DNA methylation has been also documented in hematological malignancies [2], as the regulation of CpG methylation has been established as a crucial event for stem cells and their differentiation potential. In this perspective, the analysis of DNA methylation status may be useful to identify tumor markers and therapeutic targets in cancer patients.

According to the Human Genome Assembly GRCh37, 28,299,634 CpG islands have been annotated, and up to 25% of them are located within Alu elements [3], belonging to the Short Interspersed Repetitive Elements (SINEs) class. Alu elements are relatively rich in CpG sites, and so undergo ample methylation. Interestingly, due to their prevalent localization in gene-rich regions, epigenetic alterations in Alu sequences may directly affect gene regulation in both normal and pathological conditions [4].

Methylation of Alu repeats is variable in different tissues and it is widely known that it is decreased in several types of cancer. Alu sequences have been demonstrated to contribute to establish the epigenetic landscape of cancer cells, and several papers have been focused on this topic [5–7].

In hematological malignancies, global aberrant DNA methylation has been widely documented in terms of impact on identification of leukemia molecular subtypes, disease progression and response to therapy [1, 8, 9].

To date, methylation status of Alu sequences or other DNA repeats has also been investigated [10–12] by applying different methods already in use for global DNA methylation analysis. A relationship between global DNA hypomethylation and chromosomal instability has also been highlighted in carcinogenesis [13, 14]; genomic instability, in turn, plays a major role in solid and hematological malignancies [15].

In the era of cancer epigenetics, Alu methylation investigation may be important not only to evaluate the global DNA methylation variations in disease, and the impact of Alu epigenetic variations on gene expression and disease development, but also for the molecular monitoring of cancer therapies based on hypomethylating agents. In this perspective, the molecular effects of the hypomethylating drugs decitabine (DEC) and 5-azacytidine (AZA), used to treat some hematological malignancies such as acute myeloid leukemia (AML) and myelodysplastic syndromes (MDS), could be investigated, as already reported in some previous studies [10]. Based on these considerations, Alu repeats are a good candidate as a surrogate reporter of methylation status for the entire genomic DNA of an organism owing to their homogeneous distribution throughout the human genome.

Nowadays, there are a wide variety of assays commonly applied for the evaluation of genome-wide DNA methylation, but none of them is currently performed in clinical practice; in fact, the response to hypomethylating therapy is currently based only on clinical parameters [16, 17].

In this work, we introduce the use of droplet digital PCR (ddPCR) for the evaluation of Alu repeats methylation status. In details, we suggest technical improvements to QUAlu (Quantification of Unmethylated Alu) [18] approach, a quantitative PCR technique consisting in the quantification of Unmethylated Alu repeats encompassing CpG dinucleotides, after digestion of genomic DNA with Alu-in/sensitive isoschizomers. QUAlu is based on Real Time PCR approach, that we propose to replace with ddPCR.

ddPCR is a direct method for the precise and absolute quantification of nucleic acids, based on limiting partition of the PCR reaction volume and on Poisson statistics [19, 20]. The two approaches differ in two main points: the partitioning of the PCR reaction into thousands of individual reactions prior to amplification, and the acquisition of data at reaction end point. These factors offer the advantage of direct and independent quantification of DNA without standard curves, and allow to obtain more accurate and reproducible data versus Real Time PCR [21, 22].

We therefore propose a ddPCR assay to quantify Alu sequences methylation level, and tested it on samples from patients affected by hematologic malignancies, either to verify the methylation status, or to measure and monitor Alu methylation level before and after hypomethylating treatment in hematologic malignancies.

## Methods

### Patients

This study included a total of 46 patients affected by hematologic malignancies, subdivided into three groups: a) thirty patients affected by chronic lymphocytic leukemia (CLL) (Twenty-three males and seven females; median age at diagnosis 59 years, range 28–79 years); b) seven patients affected by myelodysplastic syndromes (MDS), at intermediate/high risk according to the International Prognostic Scoring System (IPSS) score [23] (six males and one female, median age at diagnosis 68 years, range 61–81 years); c) nine patients with chronic myelomonocytic leukemia (CMML) (six males and three females, median age at diagnosis 71 years, range 62–83 years). For CLL patients, the presence of the 11q, 13q and 17p deletions, and trisomy 12 (del(11q), del(13q), del(17p), and + 12, respectively), was identified by Fluorescent In Situ Hybridization (FISH), as previously reported [24, 25]. CLL patients without the above-mentioned alterations were globally classified as “normal karyotype”. The 13q14.3 deletion was detected as the sole cytogenetic alteration in 6/18 (33%) patients, whereas in the remaining cases it was present in association with del(17p) (28%), del(11q) (28%), + 12 (22%) and del(6q) (5%). Del(11q) and del(17p) were present as the sole cytogenetic abnormality in #18, #12 and #29 cases, respectively. CLL patients were also analyzed for mutational status of the IgV_H_ and NOTCH1 gene hotspot c.7541_7542delCT by Sanger Sequencing and allele-specific oligonucleotide PCR (ASO-PCR), respectively. For MDS patients, bone marrow (BM) samples were analyzed at diagnosis and during AZA treatment. CMML patients at diagnosis were previously profiled for mutations in ASXL1 exon 12 and the SRSF2 hotspot gene mutation (c.284C>D) by SS and ASO-PCR, respectively.

The most important patients’ clinical and molecular characteristics are summarized in Additional files 1-3: Tables S1-S3. For MDS patients, the number of AZA cycles received up to the time of BM aspiration is indicated (Additional file 2: Table S2). Ten healthy donors (HD) were included in our analysis as controls (six males and four females, median age 58 years, range 55–81 years). This study was approved by the local ethics committee, and all patients provided written informed consent to take part in this project.

### Cell lines samples

Acute promyelocytic leukemia (APL)-derived NB4 cells (DSMZ, Braunschweig, Germany) were also included in the study. As previously described, the PML/RARα translocation in APL is associated with an overall increase in methylation [26], as well as in untreated NB4 cells [27]. Cells were plated using 2 normal T25 culture flasks (1.5 × 10^7^ cells in each flask) with 15 mL of RPMI 1640 supplemented with 10% heat inactivated FBS, 1% penicillin/streptomycin and 375 μl DEC 30 μM (final concentration of 0.75 μM) or 375 μL of PBS, and were incubated for 3 days at 37 °C, 5% CO_2_. Four experiments of our ddPCR assay on NB4 cultures, untreated or treated with DEC, were done on four different days. DEC concentration used in cellular experiments were compliant with the maximum concentrations reached in human plasma at clinically routine dosages [28, 29].

### Sample preparation and digestion/ligation reactions

Genomic DNA (gDNA) was directly isolated from peripheral blood (PB) using the QIAamp DNA Blood Mini Kit (Qiagen, Hilden, Germany), and from BM and cell cultures (collected cells were first resuspended in RLT buffer and PBS solution, respectively) using the DNeasy Blood & Tissue Kit (Qiagen). Extracted gDNA samples were quantified with the Qubit 2.0 Fluorometer (Thermo Fisher Scientific, Waltham, MA).

According to the manufacturer’s digestion-ligation procedures (EpiJET DNA Methylation Analysis kit, T4 DNA Ligase, Thermo Scientific), we used 250 ng of gDNA input for each reaction. For each DNA sample two aliquots of 250 ng of gDNA were simultaneously digested with 1 unit of either MspI or HpaII restriction enzymes, and ligated to a previously prepared synthetic adaptor [18] in parallel in two separate tubes. The synthetic adaptor was previously prepared [18] by incubating two complementary oligonucleotides at 65 °C for 2 min, and then at 18 °C for 35 min.

In the digestion-ligation reaction, 250 ng of gDNA, 1 μL of a synthetic adaptor 0.1 μM, 1 μl of MspI or HpaII (corresponding to 1 U), and 2 U of T4 ligase were added, in addition to 2 μl of ligase buffer 10X and 2 μl of MspI/HpaII buffer 10X, and nuclease-free water to a final volume of 40 μl. Thermal conditions for digestion and ligation were: 1 h at 37 °C and 2 h at 16 °C.

The digestion-ligation mixtures (final concentration 6.25 ng/μl) were subsequently serially diluted through sequential dilutions to obtain a final concentration of 2 pg/μl corresponding to a final amount of 6 pg distributed in three wells.

Ten samples were diluted to two final concentrations, 10 pg/μl and 2 pg/μl, to test the use of smaller amounts of DNA samples and evaluate the reproducibility of Alu sequences methylation data starting from different amounts of digested/ligated gDNA. One single test was executed on patients’ samples, because the initial amount of gDNA was the limiting factor.

### ddPCR reaction and data analysis

Like QUAlu [18], this assay is based on the selective amplification of Alu sequences containing a CpG site within the Alu consensus sequence AACCCGG.

We defined the precise amount of gDNA as input of our Alu assays using serial diluted mixtures, to obtain a final concentration of 10 pg/μL or 2 pg/μL, so as to get smaller amounts of template and to avoid the droplet saturation limit in ddPCR. For each sample the two final dilutions of digestion-ligation mixtures of MspI and HpaII were analyzed.

Prior to proceeding further with ddPCR tests, we verified that the primers used did not form any dimers during the amplification step nor aspecific sequences, by performing qualitative PCR.

ddPCR experiments were performed using the QX-200 instrument (Bio-Rad, Hercules, CA). ddPCR experiments were conducted in triplicate for both the digestion-ligation mixtures. The 20 μL ddPCR reaction mixture was then loaded into the Bio-Rad DG8 droplet generator cartridge. A volume of 70 μL of droplet generation oil was loaded for each sample. The cartridge was placed in the QX200 droplet generator. Thermal-cycling conditions were 95 °C for 5 min (1 cycle), 95 °C for 10 s (ramp rate 2 °C/second, 40 cycles), 65 °C for 7 s (ramp rate 2 °C/second, 40 cycles), 4 °C for 5 min (ramp rate 2 °C/second, 1 cycle), 90 °C for 5 min (ramp rate 2 °C/second, 1 cycle), and 4 °C hold. The expected amplicon size was 57 bp.

After amplification, the 96-well PCR plates were loaded on the Bio-Rad QX200 droplet reader and ddPCR data were analyzed with QuantaSoft analysis software (version 1.7.4, Bio-rad). Since the amplicon size was slightly below the range generally recommended in ddPCR, for wells flagged as “No Call” visual inspection and manual setting of the threshold value was performed. The target concentration in each sample was expressed as digested consensus Alu copies/μL.

Considering that the genomic DNA amount in a human diploid cell is about 6 pg/cell, for each sample we calculated the percentage of methylated consensus Alu sequences as the ratio between the sum of positive droplets obtained from the three wells of both HpaII (MH) and MspI (MM) final dilutions, according to the following formula: *[1-(sumMH/sumMM)]× 100*.

### Statistical analysis

Statistical analysis was performed using conventional pipelines in R 3.1.2 (ww.r-project.org). To test for differences between two distributions, the Wilcoxon Rank Sum Test for matched (MDS patients) or independent samples was used, whereas the Kruskal-Wallis and Dunn tests were used for comparing more than two groups of patients. Spearman correlation was used for correlation analysis. The significance level was set at *p* < 0.05 for all analyses.

### Genomic distribution and annotation analysis of Alu consensus sequences

According to the GRCh37/hg19 human genome assembly, genomic coordinates and fasta sequences of Alu repeats were retrieved from UCSC Genome Browser (https://genome.ucsc.edu/index.html). Alu consensus sequences were retrieved by searching the Alu consensus motif AACCCGG. ChIPseeker and org.Hs.eg.db R packages were used for genomic distribution analysis of Alu consensus sequences. Since our ddPCR assay involved Alu sequences containing the AACCCGG consensus motif, we retrieved Alu genomic coordinates and fasta sequences, and filtered those containing this motif.

## Results

### Genomic distribution of Alu consensus sequences

From a total of 1,142,278 Alu sequences, 171,702 (about 15%) had the consensus sequence and were the real target of our assay. Considering a region range of Transcription starting site (TSS) of 3000 bp, 74.4% of Alu sequences were annotated as “distal intergenic”, and about 5% were in the promotor regions (Additional file 4: Table S4).

We also observed a positive correlation between the chromosome length and Alu sequences distribution (rho Spearman correlation = 0.74, *p* = 5.64 × 10^− 5^), indicating a globally homogeneous distribution of these target sequences along all the human chromosomes.

### Testing of Alu methylation data reproducibility

**Fig. 1:**
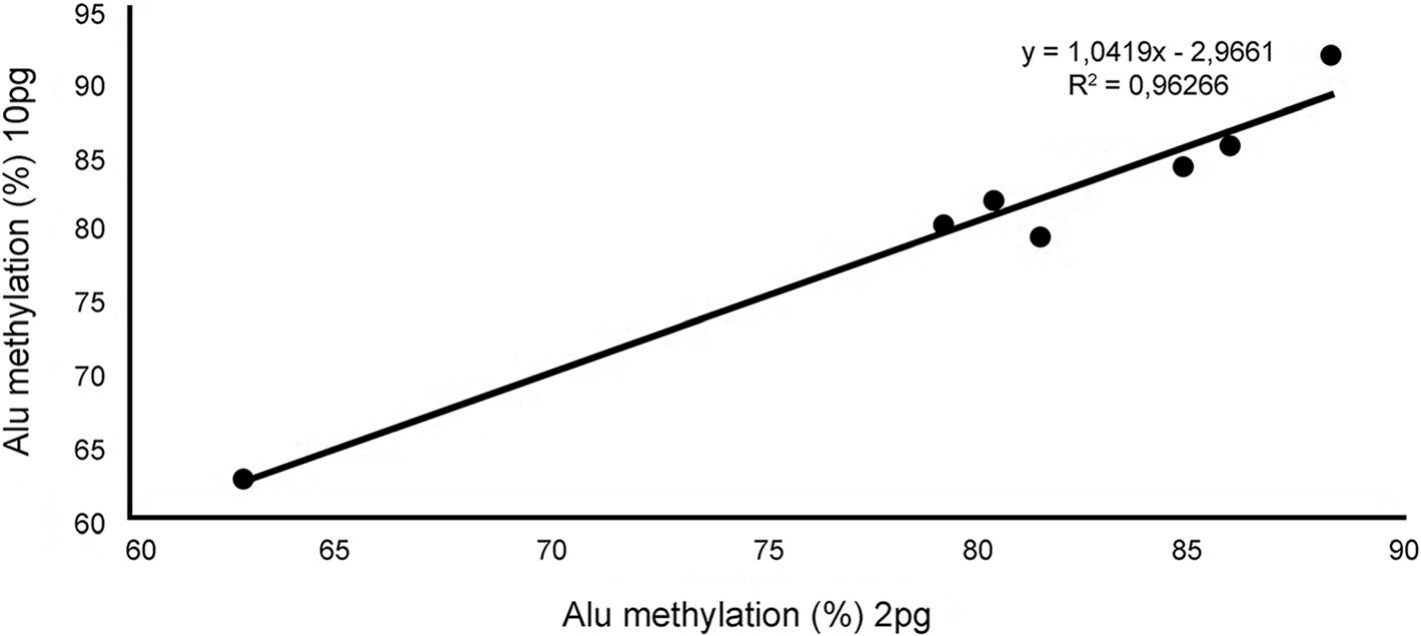
Coefficient correlation of Alu methylation levels in different amounts of gDNA (10 or 2 pg of genomic DNA for well) of 7 of 10 CLL samples. For 3 CLL samples, positive droplets saturation was observed starting from 10 pg

Comparing the Alu methylation level of 7 out of 10 CLL patients, we did not observe any statistically significant difference starting from 10 or 2 pg/μL (*p* = 0.6). As observed in Fig. 1, the Alu methylation assay by ddPCR starting from 10 or 2 pg for well showed a good linearity (R^2^ = 0.9627); however, in 3 out of 10 cases analyzed, we observed droplet saturation starting from 10 pg of gDNA for well. Indeed, QuantaLife software was not able to discriminate between positive and negative results and returned “No Call” for wells with too many positive droplets. For this reason, we decided to perform all the subsequent ddPCR experiments using 2 pg of digested/ligated gDNA for each ddPCR reaction, and calculated the sum of the three values of positive droplets.

### Alu methylation levels in healthy donors and CLL samples

**Fig. 2:**
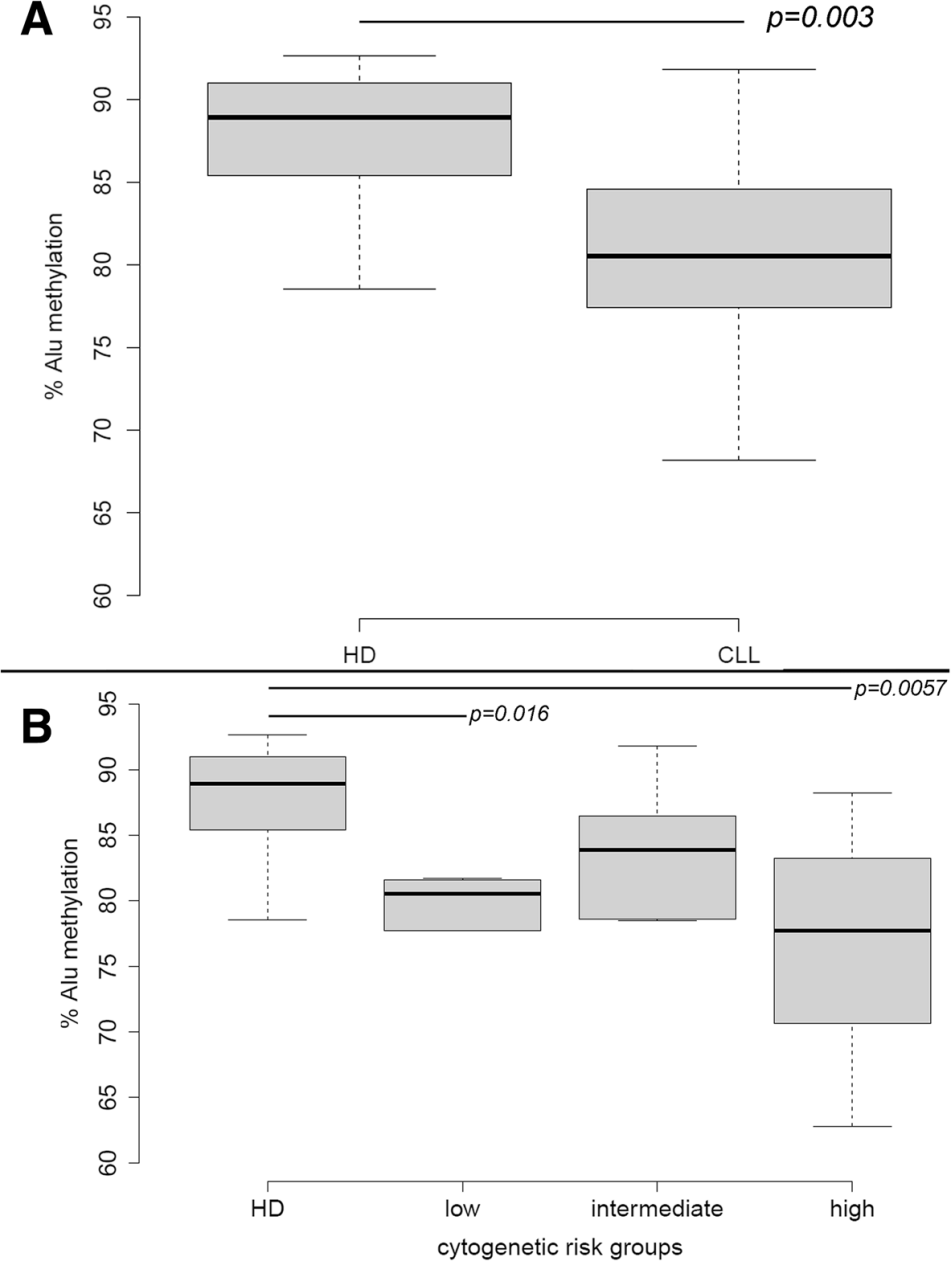
Alu methylation analysis in CLL patients. **a** Alu methylation level in CLL patients compared with HD, and (**b**) in relation to different cytogenetic risk groups according to the karyotypic alterations identified by FISH. Only the statistically significant differences (*p* < 0.05) are indicated. HD, healthy donor; CLL, chronic lymphocytic leukemia

We assessed our assay in CLL patients at diagnosis. ddPCR data from 30 CLL patients were compared with the values obtained from 10 age-matched HD, showing a statistically significant decrease of Alu methylation in CLL patients compared to the HD values (*p* < 0.05, Fig. 2a).

We also investigated the global Alu methylation level in relation to different cytogenetic risk groups. To this aim, CLL patients were classified in the following three groups according to the karyotypic alterations identified by FISH: low-risk (with isolated del(13q)), intermediate (with normal karyotype or + 12), and high risk (with del(11q), del(17p) or more than two chromosomal aberrations detected by FISH analysis). For CLL patients harboring two cytogenetic aberrations, the highest-risk alteration observed was considered [30]. Alu methylation status of the low-risk and high-risk groups was significantly reduced compared to HD (*p* < 0.05), whereas considering intermediate-risk patients the difference was not evident (Fig. 2b).

We also extended the analysis to other prognostic factors such as immunoglobulin heavy-chain variable (IgV_H_) mutational status and the presence of NOTCH1 hotspot c.7541_7542delCT, but no statistically significant difference in ALU methylation levels was observed according to these molecular parameters (data not shown).

### NB4 cell line

**Fig. 3:**
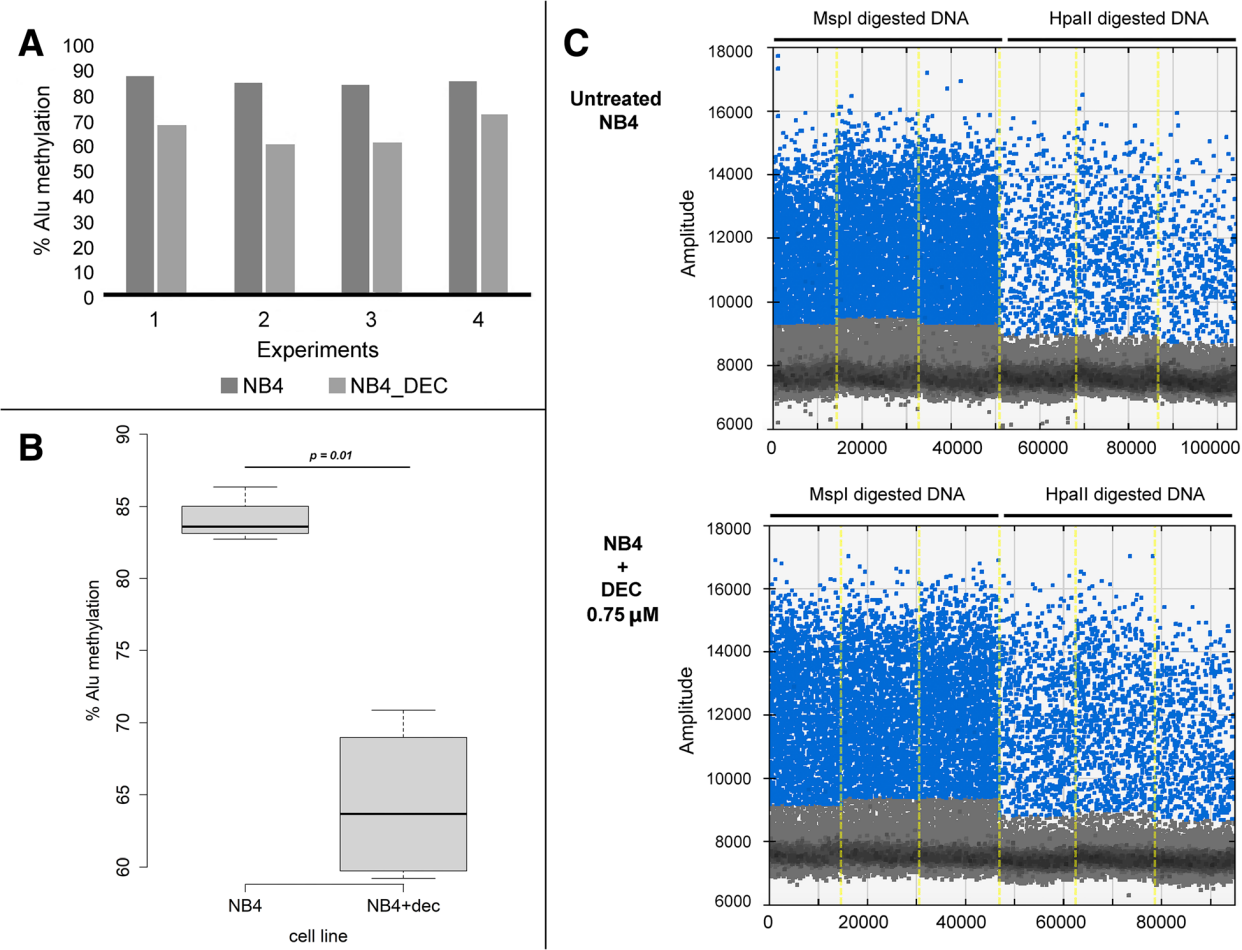
Methylation analysis with ddPCR assay in NB4 cell line. **a** Methylation level analysis of the NB4 cell line in the four experiments. **b** Box plot representation of DNA methylation levels in the untreated NB4 cell line or post treatment with hypomethylating agent. **c** ddPCR assay for the NB4 cell line in two different conditions: untreated or treated with DEC 0.75 uM. The percentage of methylated consensus Alu sequences is calculated as the ratio between the sum of the three values of positive droplets compared to HpaII (MH) and MspI (MM) diluted digestion-ligation mixtures. DEC, decitabine

We performed four experiments of our ddPCR assay on the gDNA extracted from NB4 cultures (Fig. 3a), and compared the global Alu methylation level of untreated cells to the levels in those treated with DEC hypomethylating agent. The mean of the four experiments was 84.07 and 64.35 for untreated and DEC treated cells, respectively (standard deviation, SD = 1.58, and 7.21, respectively). We observed a significant decrease of the global Alu methylation level in DNA extracted from NB4 cells treated with DEC 0.75 μM, as compared to untreated cells (*p* < 0.05) (Fig. 3b). In Fig. 3c a replicate of our ddPCR assay performed on the NB4 cell line in the two different conditions is shown.

These data suggested the potential ability of ddPCR to successfully detect global Alu methylation variations, and its potential use in the molecular monitoring of onco-hematological patients undergoing hypomethylating treatment.

### MDS patients treated with hypomethylating agents

On the basis of the preliminary results obtained analyzing NB4 cells, we decided to test our ddPCR assay on genomic DNA from MDS patients treated with AZA.

**Fig. 4:**
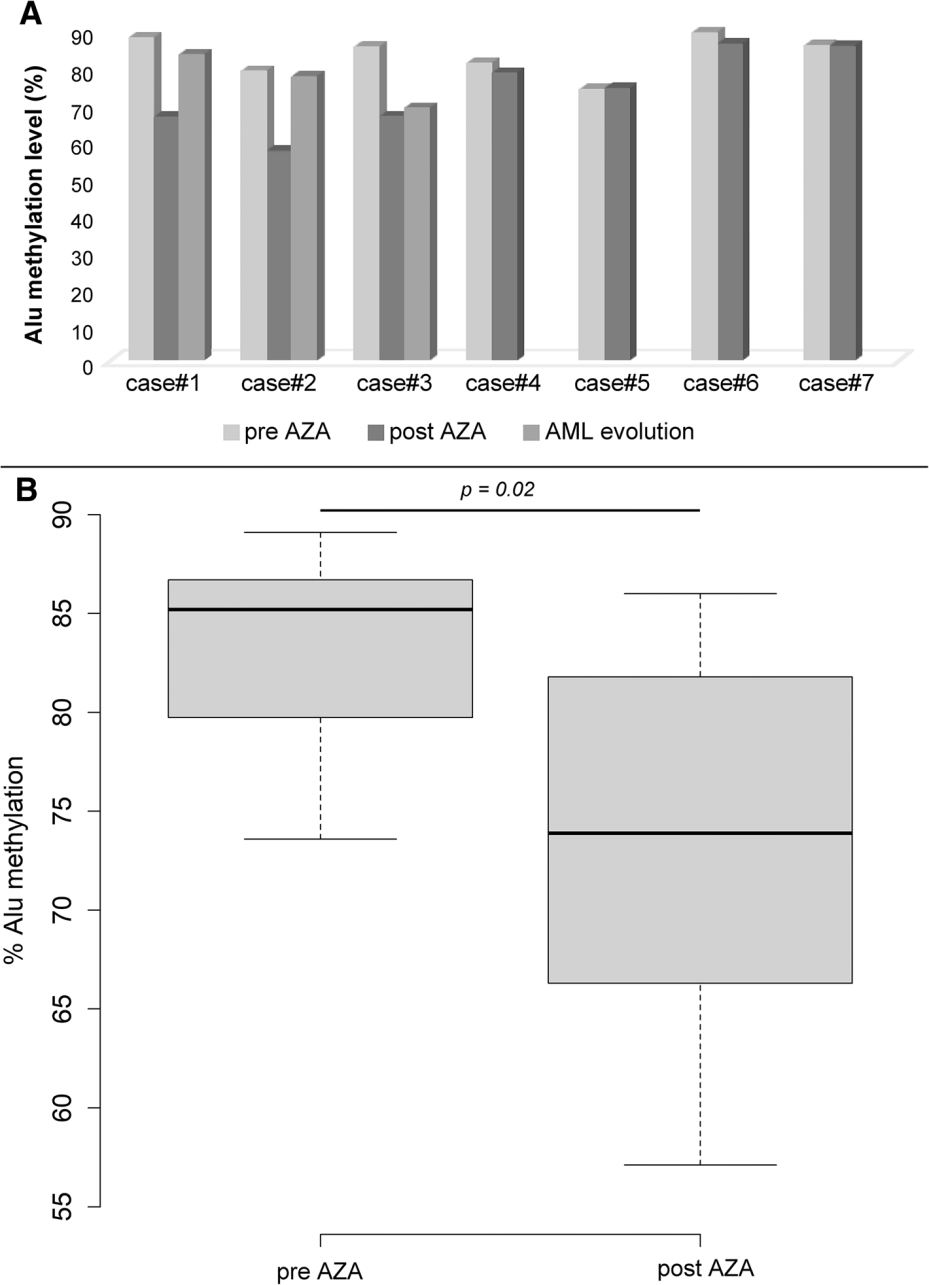
Alu methylation analysis of MDS patients. **a** Alu methylation levels of the 7 MDS patients analyzed at diagnosis and after the cycles with AZA. For 3 MDS patients (cases#1–3), the methylation level was also analyzed after the progression to AML. **b** Box plot representation of Alu sequences methylation levels in relation to hypomethylating agents therapy. The percentage of methylated consensus Alu sequences was calculated as the ratio between the sum of the three values of positive droplets compared to HpaII (MH) and MspI (MM) diluted digestion-ligation mixtures. AZA, 5-azacytidine

Paired samples from 7 MDS patients at diagnosis and after some cycles with AZA were retrospectively analyzed according to the availability of BM samples (Additional file 2: Table S2). Comparing the global paired Alu methylation levels at diagnosis and after AZA treatment, we observed a statistically significant decrease of Alu sequences methylation after therapy (*p* < 0.05) as compared to diagnosis (the median Alu methylation level was 73 and 85% for pre- and post-treatment patients, respectively; Fig. 4a-b). Secondary AML from MDS occurred in 3 patients (Case #1–3), probably correlating with the observation of a new increase of the global Alu methylation level (Fig. 4a).

### CMML patients

Finally, we attempted to test the assay in 9 CMML patients; 2 patients were mutated for ASXL1 and 2 for SRSF2 (exon 12 and the c.284C>D hotspot, respectively; Additional file 3: Table S3), two frequently mutated genes in CMML [31–33].

**Fig. 5:**
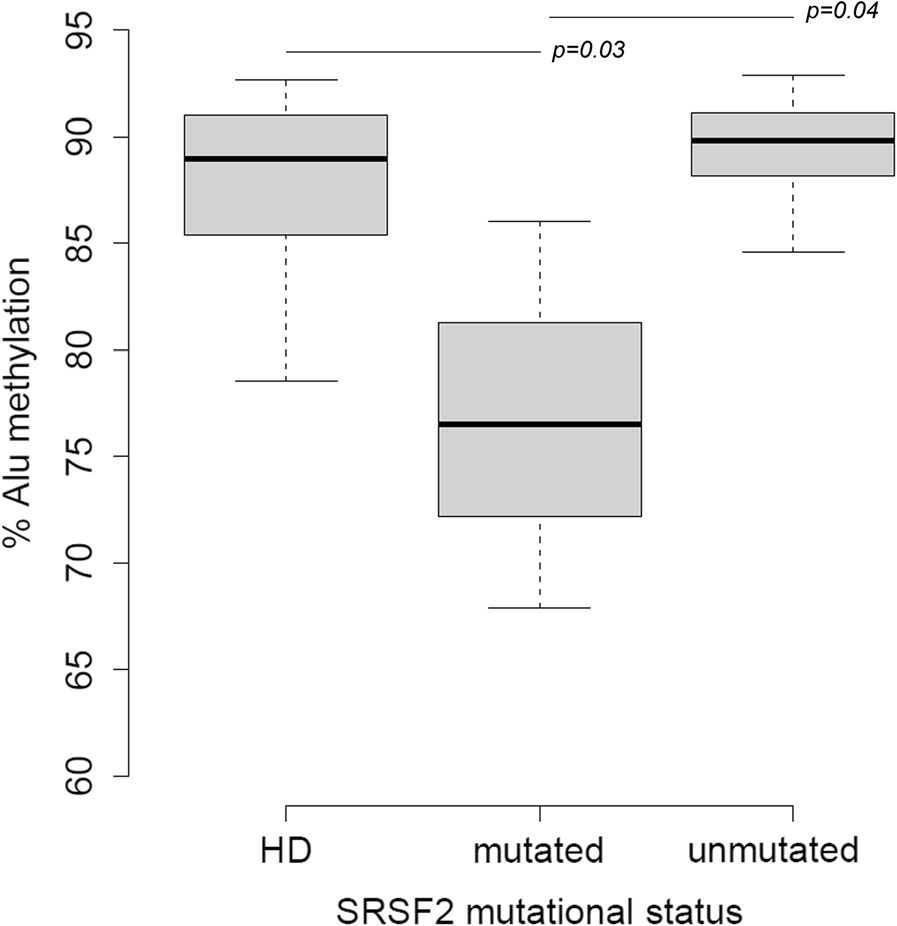
Alu DNA methylation levels in CMML patients. Alu methylation analysis of a small cohort of CMML patients compared with HD, and in relation to the presence of the SRSF2 hotspot (c.284C>D). HD, healthy donor

Overall, CMML patients showed no differences in Alu methylation levels as compared with HD (*p* > 0.05). Among CMML patients, a decrease of Alu sequences methylation was observed in those harboring the main SRSF2 hotspot compared to patients without this mutation (*p* < 0.05, Fig. 5), whereas no significant difference was observed according to the presence of mutations in ASXL1 exon 12 (data not shown).

## Discussion

Alu elements contain about 25% of all CpGs of the human genome, reside mainly in gene-rich regions and are therefore more suitable for evaluating the global methylation than other repetitive DNA elements [34–37]. It is likely that Alu demethylation contributes to affect the regulation of nearby genes. However, it is not known if Alu methylation could be the driving force for nearby gene expression variations or, alternatively, if Alu methylation is influenced by other nearby genomic features [38].

Methylation of Alu elements varies in different tissues and seems to be decreased in many types of cancer; indeed, global aberrant DNA methylation is described as a crucial epigenetic alteration in several solid and hematological malignancies [1, 9]. For example, Chen et al. hypothesized that methylation patterns of Alu in serum could be considered a diagnostic and prognostic factor for glioma patients. They used microsphere arrays that work by recognition of 5-hydroxymethylcytosine or thymidine in CpG islands in order to determine the methylation status of Alu, finally demonstrating a significantly lower methylation levels of Alu in high-grade compared with low-grade glioma [6]. An association between global DNA hypomethylation and genomic instability leading to cancer development has also been reported [13, 15]; in this context, investigating global DNA methylation may be interesting in view of the relevant role of genomic instability in hematological malignancies [39] .

In the last years, a wide variety of techniques has been designed to measure or predict global DNA methylation, some of which are focused on the methylation level of specific genomic compartments, especially repeat elements [40, 41]. Some previous studies attempted to investigate Alu methylation even in hematological malignancies [10–12, 42], by applying several methods; overall, these approaches are laborious and time-consuming.

In our work we propose ddPCR technology, which represents an alternative to conventional quantitative-PCR (qPCR) for the quantification of DNA templates [43, 44]. The key of Poisson statistics lies in optimizing the number of positive droplets (positive events) to the total number of droplets (independent events). To this aim, calculating the sum of positive droplets in about 6 pg of DNA, with 20,000 droplets generated by our ddPCR system, an accurate starting template estimation is guaranteed. In the case of “No Call” results with evident positive droplets, a unique threshold was not a priori established; in the next future, further progress may be useful to overcome this limit.

This assay is based on the QUAlu method [18], but introduces ddPCR technology as a more sensitive technique. Studies focused on the use of ddPCR in hematological malignancies have previously demonstrated a more precise molecular target quantification in comparison with RT-qPCR [45–47]. The use of ddPCR to detect DNA methylation has recently emerged and adopted in cancer field [48, 49], but these approaches focus on specific CpG sites or targets. To the best of our knowledge, this is the first work that describes the potential application this technology for assessing global DNA methylation by inspecting short DNA repeats.

Our findings demonstrate some advantages of using ddPCR for Alu methylation analysis: firstly, it may be carried out using very small amounts of digested gDNA (about 6 pg corresponding to the gDNA amount in a human diploid cell), and a reference gene is not needed for the analysis. It is also easy to perform and does not require complex statistics or bioinformatic analysis. By using different initial amounts of gDNA template (2–10 pg) the results were consistent, suggesting reproducibility and sensitivity.

The main intent of the study is to evaluate the feasibility of Alu methylation status by ddPCR, rather than to demonstrate the specific relationship between Alu methylation and specific onco-hematologic patients’ groups. Although the work does not have the aim to demonstrate a link between the pathogenesis of blood neoplasia and methylation, some observations arising from our results can be made. The possibility of detecting changes in the global methylation before and after exposure to the hypomethylating agent by ddPCR suggests that it may be useful to monitor the effectiveness of treatment. We observed a global Alu methylation decrease by comparing paired gDNA samples from pre- and post-AZA treated patients; in this scenario, individual responses to drugs may vary or not be significant, as observed for some MDS patients analyzed (Fig. 4a).

In CMML, mutations in genes coding for chromatin modulators (ASXL1 about 40%) and spliceosome components (SRSF2 about 50%) have been shown to negatively affect patients’ prognosis, but their relationship with global DNA methylation is still unknown [50]. The preliminary observation that CMML cases associated with the SRSF2 gene mutation may be associated with a low Alu methylation profile requires to be confirmed by increasing the number of samples. However, in this context the abnormal RNA splicing may have functional consequences for methylation in cancer by generating truncated isoforms of genes involved in methylation pathways [51].

As regards CLL, repetitive elements methylation has been previously evaluated with different methods [42]. Our approach confirmed that there is a significant difference between CLL patients compared to HD, as previously observed [42]; moreover, inspecting the global methylation according to cytogenetic-risk groups, a more evident Alu methylation reduction in both low- and high-risk CLL patients was observed compared to HD. These observations are partially overlapping with previous results demonstrating the global Alu methylation level in CLL harboring del(17p) [42, 52]; this may be due to a small number of patients analyzed for single cytogenetic risk groups.

Since leukemias are often associated with chromosome instability and rearrangement events, and Alu methylation prevents genomic instability, evaluating global Alu methylation level by ddPCR may be interesting to inspect the correlation between the two molecular events.

## Conclusions

In summary, we demonstrate that ddPCR-based assay may be useful for inspecting the global DNA methylation of Alu repeats, in hematological malignancies and investigating possible epigenetic alterations for diagnostic/prognostic purposes.

## Additional files

Additional file 1: **Table S1.** Clinical characteristics of the CLL patients included in the study. (DOCX 13 kb)

Additional file 2: **Table S2.** Clinical characteristics of the MDS patients retrospectively analyzed in this study. The number of hypomethylating therapy cycles at which the analysis is performed varied according to BM sample availability. (DOCX 14 kb)

Additional file 3: **Table S3.** Molecular characteristics of the CMML patients retrospectively analyzed in this study. (DOCX 12 kb)

Additional file 4: **Table S4.** Genomic distribution of Alu consensus sequences, considering a Transcription starting site (TSS) range of 3000 bp. (DOCX 13 kb)

## Abbreviations

AML: acute myeloid leukemia
ASO-PCR: allele-specific oligonucleotide PCR
AZA: 5-azacytidine
BM: bone marrow
CLL: chronic lymphocytic leukemia
CMML: chronic myelomonocytic leukemia
ddPCR: droplet digital PCR
DEC: decitabine
FISH: Fluorescent In Situ Hybridization
gDNA: genomic DNA
HD: healthy donor
IPSS: International Prognostic Scoring System
MDS: myelodysplastic syndromes
MDS: myelodysplastic syndromes
PB: peripheral blood
QUAlu: Quantification of Unmethylated Alu
SINEs: Short Interspersed Repetitive Elements
