# Supplementary material for: Droplet digital PCR for the quantification of Alu methylation status in hematological malignancies
Source: Diagn Pathol. 2018 Dec 22;13:98. doi: 10.1186/s13000-018-0777-x (PMC6303857; doi:10.1186/s13000-018-0777-x)
Supplement: Supplementary file 1 — Table S1. Clinical characteristics of the CLL patients included in the study. (DOCX 13 kb) [file 13000_2018_777_MOESM1_ESM.docx]

**Additional File 1: Table S1.**

| **Case** | **Sex/Age** | **FISH** | **Cytogenetic-risk group** | **IgHV status** |
| --- | --- | --- | --- | --- |
| **#1** | M/28 | normal | intermediate | unmutated |
| **#2** | F/69 | 13q- | low | mutated |
| **#3** | F/64 | 13q-;17p- | high | unmutated |
| **#4** | M/66 | 13q-; 17p- | high | mutated |
| **#5** | F/58 | 13q-;11q- | high | unmutated |
| **#6** | M/70 | normal | intermediate | unmutated |
| **#7** | F/71 | 13q- | low | unmutated |
| **#8** | M/57 | 13q-;17p- | high | unmutated |
| **#9** | F/70 | 13q-;17p- | high | unmutated |
| **#10** | M/65 | 13q-;11q- | high | unmutated |
| **#11** | M/49 | 13q-;11q- | high | unmutated |
| **#12** | M/58 | 17p- | high | unmutated |
| **#13** | M/57 | normal | intermediate | mutated |
| **#14** | M/62 | +12 | intermediate | mutated |
| **#15** | M/79 | +12;13q- | intermediate | mutated |
| **#16** | M/60 | 13q- | low | mutated |
| **#17** | M/51 | +12 | intermediate | unmutated |
| **#18** | F/59 | 11q- | high | unmutated |
| **#19** | M/62 | 13q- | low | mutated |
| **#20** | M/53 | 13q- | low | mutated |
| **#21** | M/50 | +12;13q- | intermediate | mutated |
| **#22** | M/56 | +12;17p-;13q-;11q- | high | unmutated |
| **#23** | M/58 | +12 | intermediate | mutated |
| **#24** | M/59 | 13q- | low | unmutated |
| **#25** | M/44 | +12;13q- | intermediate | mutated |
| **#26** | M/41 | normal | intermediate | mutated |
| **#27** | M/64 | 6q-;11q-;13q- | high | unmutated |
| **#28** | M/77 | normal | intermediate | mutated |
| **#29** | M/54 | 17p- | high | unmutated |
| **#30** | F/53 | normal | intermediate | unmutated |
