# Supplementary material for: Droplet digital PCR for the quantification of Alu methylation status in hematological malignancies
Source: Diagn Pathol. 2018 Dec 22;13:98. doi: 10.1186/s13000-018-0777-x (PMC6303857; doi:10.1186/s13000-018-0777-x)
Supplement: Supplementary file 2 — Table S2. Clinical characteristics of the MDS patients retrospectively analyzed in this study. The number of hypomethylating therapy cycles at which the analysis is performed varied according to BM sample availability. (DOCX 14 kb) [file 13000_2018_777_MOESM2_ESM.docx]

| **Case** | **Diagnosis** | **AZA cycles** |
| --- | --- | --- |
| **#1** | MDS-EB I | 15 |
| **#2** | MDS-EB I | 26 |
| **#3** | MDS-EB II | 21 |
| **#4** | MDS-EB II | 17 |
| **#5** | MDS-EB I | 48 |
| **#6** | MDS-EB I | 20 |
| **#7** | MDS-EB II | 16 |

**Additional File 2: Table S2**
