# Supplementary material for: Droplet digital PCR for the quantification of Alu methylation status in hematological malignancies
Source: Diagn Pathol. 2018 Dec 22;13:98. doi: 10.1186/s13000-018-0777-x (PMC6303857; doi:10.1186/s13000-018-0777-x)
Supplement: Supplementary file 3 — Table S3. Molecular characteristics of the CMML patients retrospectively analyzed in this study. (DOCX 12 kb) [file 13000_2018_777_MOESM3_ESM.docx]

**Additional File 3: Table S3**

| **Case** | **SRSF2 mutational**  **status** | **ASXL1 mutational**  **status** |
| --- | --- | --- |
| **#1** | mutated | unmutated |
| **#2** | mutated | unmutated |
| **#3** | mutated | mutated |
| **#4** | unmutated | mutated |
| **#5** | unmutated | mutated |
| **#6** | unmutated | unmutated |
| **#7** | unmutated | unmutated |
| **#8** | unmutated | unmutated |
| **#9** | unmutated | unmutated |
