# Supplementary material for: Droplet digital PCR for the quantification of Alu methylation status in hematological malignancies
Source: Diagn Pathol. 2018 Dec 22;13:98. doi: 10.1186/s13000-018-0777-x (PMC6303857; doi:10.1186/s13000-018-0777-x)
Supplement: Supplementary file 4 — Table S4. Genomic distribution of Alu consensus sequences, considering a Transcription starting site (TSS) range of 3000 bp. (DOCX 13 kb) [file 13000_2018_777_MOESM4_ESM.docx]

**Additional File 4: Table S4**

Genomic distribution summary of Alu consensus sequences.

| **Feature** | **Frequency (%)** |
| --- | --- |
| Promoter (<=1kb) | 1.11 |
| Promoter (1-2kb) | 1.68 |
| Promoter (2-3kb) | 1.69 |
| 5' UTR | 0.01 |
| 3' UTR | 0.32 |
| 1st Exon | 0.03 |
| Other Exon | 0.06 |
| 1st Intron | 3.6 |
| Other Intron | 17.1 |
| Distal Intergenic | 74.4 |
